# Coenzyme Q10 or Creatine Counteract Pravastatin-Induced Liver Redox Changes in Hypercholesterolemic Mice

**DOI:** 10.3389/fphar.2018.00685

**Published:** 2018-06-27

**Authors:** Ana C. Marques, Estela N. B. Busanello, Diogo N. de Oliveira, Rodrigo R. Catharino, Helena C. F. Oliveira, Anibal E. Vercesi

**Affiliations:** ^1^Departamento de Patologia Clínica, Faculdade de Ciências Médicas, Universidade Estadual de Campinas, Campinas, Brazil; ^2^Departamento de Biologia Estrutural e Funcional, Instituto de Biologia, Universidade Estadual de Campinas, Campinas, Brazil

**Keywords:** pravastatin, LDL receptor knockout mice, mitochondria, reactive oxygen species, coenzyme Q10, creatine

## Abstract

Statins are the preferred therapy to treat hypercholesterolemia. Their main action consists of inhibiting the cholesterol biosynthesis pathway. Previous studies report mitochondrial oxidative stress and membrane permeability transition (MPT) of several experimental models submitted to diverse statins treatments. The aim of the present study was to investigate whether chronic treatment with the hydrophilic pravastatin induces hepatotoxicity in LDL receptor knockout mice (*LDLr*^-/-^), a model for human familial hypercholesterolemia. We evaluated respiration and reactive oxygen production rates, cyclosporine-A sensitive mitochondrial calcium release, antioxidant enzyme activities in liver mitochondria or homogenates obtained from *LDLr*^-/-^ mice treated with pravastatin for 3 months. We observed that pravastatin induced higher H_2_O_2_ production rate (40%), decreased activity of aconitase (28%), a superoxide-sensitive Krebs cycle enzyme, and increased susceptibility to Ca^2+^-induced MPT (32%) in liver mitochondria. Among several antioxidant enzymes, only glucose-6-phosphate dehydrogenase (G6PD) activity was increased (44%) in the liver of treated mice. Reduced glutathione content and reduced to oxidized glutathione ratio were increased in livers of pravastatin treated mice (1.5- and 2-fold, respectively). The presence of oxidized lipid species were detected in pravastatin group but protein oxidation markers (carbonyl and SH- groups) were not altered. Diet supplementation with the antioxidants CoQ10 or creatine fully reversed all pravastatin effects (reduced H_2_O_2_ generation, susceptibility to MPT and normalized aconitase and G6PD activity). Taken together, these results suggest that 1- pravastatin induces liver mitochondrial redox imbalance that may explain the hepatic side effects reported in a small number of patients, and 2- the co-treatment with safe antioxidants neutralize these side effects.

## Introduction

Statins are inhibitors of the 3-hydroxy-3-methylglutaryl coenzyme-A (HMG-CoA) reductase widely used to lower plasma cholesterol levels of hypercholesterolemic patients (Endo, 1992). Besides lowering plasma cholesterol levels, other beneficial effects are claimed, including anti-inflammatory (Liao, 2002), antioxidant (Sirtori, 2014), and antitumor (Vallianou et al., 2014) properties of statins. In addition, statins seems to contribute to slow down the progression of fibrosis and reduce the incidence of hepatocellular carcinoma in cirrhotic patients (Abraldes and Burak, 2016).

On the other hand, some patients present class and dose-dependent adverse effects in muscle, liver and other tissues (Beltowski et al., 2009). Although rare, statins alone may induce liver injury (Medina-Caliz et al., 2016; Björnsson, 2017) and their toxicity can be potentiated by the combination with other drugs (Andrade et al., 2005; Chalasani et al., 2008; Björnsson and Hoofnagle, 2016). Liver autoimmune responses with varying onset latency (Reuben et al., 2010) and progression (Russo et al., 2014) may also occur.

Liver adverse symptoms are in general unspecific and most patients remain asymptomatic (Tzefos and Olin, 2011). An increase in plasma aspartate (AST) and alanine (ALT) aminotransferases that may be accompanied by bilirubin elevation (Herrick et al., 2016) was described in less than 1% of treated patients (Aguirre et al., 2013). Two factors are frequently related to the hepatotoxic effects of statins: their lipophilicity and metabolism by the cytochrome P450 system (Bhardwaj and Chalasani, 2007; Herrick et al., 2016). Accordingly, prospective studies showed an association between statins, especially lipophilic, with drug-induced liver injury (DILI). Between 1994 and 2012, 5.5% (47/858) of the DILI cases were related to statins in Spain and from 2004 to 2014, 1.9% (22/1188) cases were reported in United States (Björnsson, 2017). Rare cases of portal inflammation or fibrosis that can be followed by necrosis were also described in patients under lovastatin (MacDonald et al., 1988) or atorvastatin treatment (Nakad et al., 1999). On the other hand, hydrophilic statins are minimally metabolized by cytochrome P450 pathway (Bhardwaj and Chalasani, 2007) and are generally less toxic (Velho et al., 2006). In fact, a multicenter report indicated that pravastatin is well tolerated in patients with compensated chronic liver disease (Lewis et al., 2007).

Our group and others attribute statins-induced liver toxicity to mitochondrial dysfunction associated with oxidative stress and mitochondrial membrane permeability transition (MPT) (Velho et al., 2006; Bhardwaj and Chalasani, 2007; Mohammadi-Bardbori et al., 2015; Tolosa et al., 2015). A recent study showed that inner mitochondrial membrane depolarization, ATP depletion and MPT occurred in liver mitochondria from normolipidemic rats fed with high fat diet and treated with simvastatin or atorvastatin for 30 days. These effects were counteracted by co-treatment with coenzyme Q10 (CoQ10) (Mohammadi-Bardbori et al., 2015). MPT triggered by Ca^2+^ and increased superoxide production were also found in HepG2 cells after incubation with several statins (Tolosa et al., 2015). We have previously shown increased MPT in liver mitochondria from hypercholesterolemic mice treated for short term (2 weeks) with the lipophilic lovastatin (Velho et al., 2006) and a mild skeletal muscle mitochondrial oxidative stress after chronic treatment (3 months) with the hydrophilic pravastatin (Busanello et al., 2017).

Considering the relevance of understanding the hepatic side effects of specific statin classes, the aim of the present work was to investigate the liver mitochondrial function and redox state in an appropriated biological context (hypercholesterolemia) under chronic treatment with moderate dose of a less toxic hydrophilic statin (pravastatin). In addition, we investigated whether CoQ10 or creatine diet supplementation protects against statins toxicity.

## Materials and Methods

### Animals and Chemical Reagents

LDL receptor knockout mice (*LDLr*^-/-^) were provided by Campinas University Multidisciplinary Center for Biological Research in Laboratory Animals (CEMIB-UNICAMP) and mice founders were purchased from The Jackson Laboratory (Bar-Harbor, ME, United States). This study was performed in accordance with the Guide for the Care and Use of Laboratory Animals published by National Academy of Sciences and with the approval of University Committee for Ethics in Animal Experimentation (protocol # 3401-1). Mice had free access to water and standard AIN/93 M diet (Prag-Soluções, SP, Brazil) and were housed in a 22 ± 2°C room with a 12 h light-dark cycle. Chemical reagents were purchased from Sigma-Aldrich (St. Louis, MO, United States) and Molecular Probes-Invitrogen (Carlsbad, CA, United States).

### Pravastatin Treatment and Antioxidants Supplementation

Thirty-day-old male *LDLr*^-/-^ mice received pravastatin (Medley Ltda, Brazil) diluted in the drinking water (400 mg/L) during 3 months as previously described (Lorza-Gil et al., 2016). The estimated pravastatin dose of 40 mg/kg body weight/day is based on the average drink consumption rate of 3.5 mL/day. Controls received filtered tap water. Additional groups of mice were treated with standard diet supplemented with 0.3% CoQ10 along with pravastatin treatment or with 2% creatine during the last 15 days of pravastatin treatment (Busanello et al., 2017). The final body weight (g) of mice did not differed among the groups: pravastatin vs. control: 22.2 ± 0.40 vs. 22.9 ± 0.43; pravastatin + CoQ10 vs. control + CoQ10: 21.6 ± 0.41 vs. 22.4 ± 0.33; and pravastatin + creatine vs. control + creatine: 22.1 ± 0.53 vs. 22.7 ± 0.44.

### Plasma Cholesterol and Transaminases Analysis

Blood samples were obtained with heparin-containing capillaries from the *LDLr*^-/-^ mice tail tip after a 12-h fasting period. Samples were centrifuged and plasma was used for total cholesterol and liver transaminases (AST and ALT) measurements using standard commercial kits (Roche-Diagnostics, Germany and InVitro, Brazil, respectively).

### Liver Histology

Liver biopsies were taken and fixed in 10% formalin. Fixed liver tissue samples were dehydrated in alcohol, embedded in paraffin and stained with hematoxylin and eosin (HE), Masson’s trichrome, and picrosirius red (PSR).

### Mitochondrial Isolation and Standard Procedure

Mitochondria were isolated from livers of *LDLr*^-/-^ mice by differential centrifugation (Velho et al., 2006). Isolation medium contained 1 mM EGTA, 250 mM sucrose, 10 mM HEPES and 1% BSA (pH 7.2). The final pellets composed by liver mitochondria were suspended in medium devoid of EGTA and BSA in a final protein concentration of 50 mg/mL. The experiments with isolated mitochondria (0.5 mg/mL) were carried out at 28°C with a standard reaction medium containing 15 μM Ca^2+^, 10 mM Hepes, 125 mM sucrose, 2 mM K_2_HPO_4_, 65 mM KCl, 1 mM MgCl_2_, and 5 mM glutamate plus 5 mM malate (pH 7.2).

### Oxygen Consumption by Isolated Liver Mitochondria

Mitochondrial respiration was measured in an oxygraph (OROBOROS oxygraph-2k, Austria) in the presence or absence of 200 μM EGTA (Busanello et al., 2017). After measuring the basal O_2_ consumption (state 2) required to sustain mitochondrial potential, ADP (300 μM) was added to stimulate oxidative phosphorylation (state 3) and respiration was followed up to reaching the non-phosphorylating respiratory state (state 4).

### Mitochondrial Reactive Oxygen Production Rates and Aconitase Activity

Mitochondrial hydrogen peroxide production was determined using 10 μM Amplex-red and 1 U/mL horseradish peroxidase. The generation of the fluorescent product resorufin (Ronchi et al., 2013) was monitored using a spectrofluorometer (Hitachi F-7000, Japan) operating at 563 nm excitation and 587 nm emission wavelengths.

Aconitase activity, a Krebs cycle enzyme highly sensitive to superoxide, was determined by the method described by Morrison (1954), using a medium containing 50 mM KH_2_PO_4_, 10% triton X-100, 0.07 mM sodium citrate, 1.3 mM manganese chloride. The reduction of NADP^+^ was measured at the wavelengths 340 (excitation) and 466 nm (emission) and expressed as μmol NADPH/min/mg protein.

### NADP-Dependent Mitochondrial Enzymes Activities

Isocitrate dehydrogenase-2, malic enzyme and glutamate dehydrogenase activities were determined in liver isolated mitochondria of *LDLr*^-/-^ mice. Absorbance changes of NADPH were monitored in the three enzyme assays as previously described by Ronchi et al. (2013). Results were expressed as mU/mg.

### Mitochondrial Ca^2+^ Uptake/Retention Capacity

Mitochondrial calcium uptake/release was measured by following the fluorescence changes in the medium containing 0.1 μM calcium green-5N hexapotassium salt using a spectrofluorometer (Hitachi F-4500, Japan) operating at 506 nm for excitation and 531 nm for emission (Ronchi et al., 2013).

### Liver Antioxidant Enzyme Activities and Oxidized Protein Content

*LDLr*^-/-^ mice were anesthetized with ketamine (90 mg/kg) plus xylazine (10 mg/kg) and their heart were perfused with NaCl 0.9% during 5 min. A portion of liver (200 mg) was rapidly removed, finely minced and homogenized in 1.8 mL of buffer (pH 7.4) containing 20 mM sodium phosphate and 140 mM KCl in ice-cold potter Elvehjem tissue homogenizer. Homogenates were then centrifuged at 1000 × *g* for 20 min at 4°C to remove nuclei and cell debris. The total supernatant was used to determine enzymatic activities and protein oxidative damage. Protein content was measured using bovine serum albumin as standard (Borges et al., 2015). Glutathione reductase (GR), glutathione peroxidase (GPx), superoxide dismutase (SOD), and catalase (CAT) were determined according to Borges et al. (2015) and peroxiredoxin (Prx) as previously described (Kim et al., 2005). Glucose-6-phosphate dehydrogenase (G6PD) activity was analyzed in a reaction mixture containing 10 mM MgCl_2_, 5 mM NADP^+^, 100 mM Tris HCl (pH 7.5) and approximately 3 μg of sample protein. The reaction started by the addition of 1 mM glucose-6-phosphate and was followed at 340 nm. One unit (U) of G6PD corresponds to 1 mmol of substrate consumed per minute and the enzyme activity is expressed as U/mg protein (Leong and Clark, 1984). Protein oxidative damage was assessed in liver supernatants by measuring carbonylated protein groups and sulfhydryl content as previously described (Borges et al., 2015). Results were expressed as nmol/mg protein.

### Glutathione Levels

Reduced (GSH) and oxidized (GSSG) glutathione levels were measured in liver homogenates by following the enzymatic recycling method according to Teare et al. (1993). The linear increase in absorbance at 412 nm over time was monitored using a microplate reader (SpectraMax M3 – Molecular Devices, United States). Experimental controls and standard curves were built with known amounts of GSH and GSSG.

### NADP^+^ and NADPH Levels

The NADP^+^/NADPH detection assay was performed in liver homogenates by using a commercial kit (Promega – NADP/NADPH – Glo Assay). The luminescence produced by the luciferin–luciferase system was accompanied in a microplate reader (SpectraMax M3 – Molecular Devices, United States). Experimental controls and standard curves were built with known concentrations of NADP^+^ and NADPH.

### Lipid Markers Determination by Electrospray Ionization High Resolution Mass Spectrometry (ESI-HRMS)

Mouse liver was homogenized with a methanol:H_2_O (50:50) solution. Resulting homogenates were filtered through a 0.22 μm nylon membrane; 10 μL of the filtrate were further diluted in a methanol:H_2_O (50:50) solution containing 0.1% formic acid to a final volume of 1 mL. Samples were directly injected in an ESI-LTQ-XL Orbitrap Discovery instrument (Thermo Scientific, Bremen, Germany). Typical operating conditions were as follows: sheath gas at 10 arbitrary units, 4.5 kV and *m/z* range of 50–500 in the negative ion mode. The matrix of mass vs. intensity derived from spectral data was submitted to orthogonal partial least squares discriminant analysis (OPLS-DA) using MetaboAnalyst 3.0 (Xia et al., 2015) to identify markers for each condition. Data preprocessing was performed, using quantile normalization, log transformation and range scaling. The significance of the model was further assessed by using permutation tests of prediction accuracy during training and separation distance. Ten of the most relevant ions for each group were selected by their scores of variable importance in projection (VIP) provided by the model. These ions were ultimately researched in the Lipid Maps database, where species of interest were identified. Structural elucidation was carried out using mass accuracy, with a mass shift (error) limit of 2 ppm or below.

### Statistical Analysis

Results are presented as representative traces and mean ± standard deviation of at least five independent experiments. Data were analysed using Mann–Whitney test. Differences between groups were rated significant at *P* ≤ 0.05. All analyses were carried out using the GraphPad software.

## Results

### Plasma Aminotransferases and Liver Histology Are Not Altered in Pravastatin Treated *LDLr*^-/-^ Mice

After 3 months of pravastatin treatment (40 mg/kg/day), plasma cholesterol levels were significantly reduced compared to control untreated *LDLr*^-/-^ mice (357.4 ± 12.2 and 407.3 ± 12.4, respectively, *P* < 0.05). No significant alterations were found in plasma levels of liver transaminases AST (28.22 ± 7.30 and 39.40 ± 7.20) and ALT (18.52 ± 5.33 and 20.59 ± 8.29) in control and pravastatin treated *LDLr*^-/-^ mice, respectively. Liver histological analyses after staining with HE, Masson’s trichrome and picrosirius red (PSR) show normal morphology and absence of steatosis, inflammation and fibrosis in the livers of *LDLr*^-/-^ mice under pravastatin treatment (**Supplementary Figure S1**).

### Mitochondrial Oxygen Consumption Rates and Coupling do Not Differ in Liver Mitochondria From Control and Pravastatin Treated *LDLr*^-/-^ Mice

**FIGURE 1:**
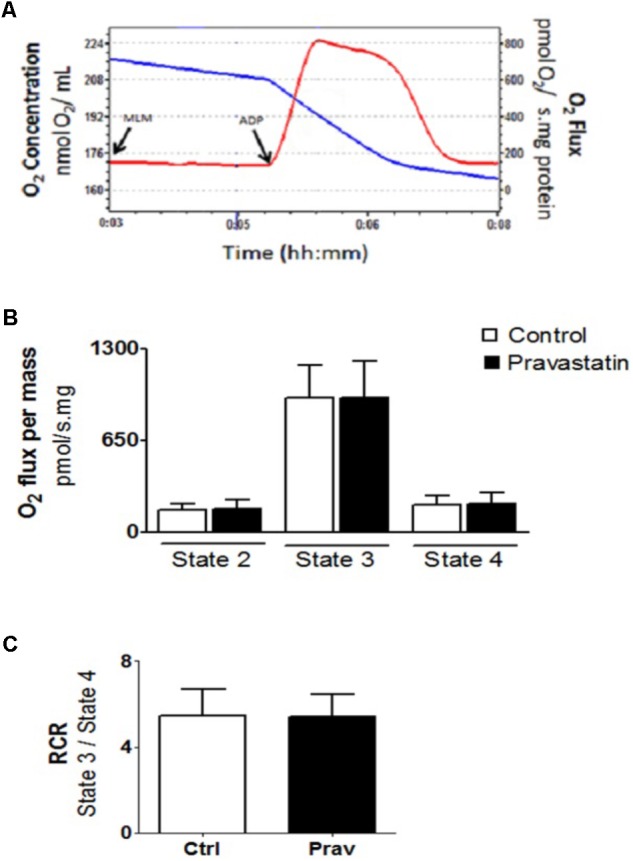
Pravastatin treatment does not alter oxygen consumption by liver mitochondria from *LDLr*^-/-^ mice. Respiration was evaluated in a standard medium containing 10 mM glutamate plus 5 mM malate, in the presence of EGTA (200 μM). ADP (300 μM) was added as indicated. Representative traces of liver mitochondria respiration showing O_2_ medium concentration (blue line), expressed as nmol O_2_/mL and O_2_ flux (red line), expressed as ρmol O_2_/s.mg protein **(A)**. Bar graphs show the rates of respiration in each state **(B)**. Respiratory control ratio (state 3/state 4, RCR) **(C)**. Values are means ± standard deviation, 10 independent experiments and expressed as ρmol O_2_/s.mg protein. No significant differences were observed (Mann–Whitney test).

The integrity of liver mitochondria isolated from *LDLr*^-/-^ mice under chronic pravastatin treatment was assessed by measuring respiration rates in the presence of EGTA (200 μM) and after addition of 300 μM ADP during the experiments. **Figure 1** shows that pravastatin treatment did not affect the mitochondrial respiratory rates in state 2 (basal), state 3 (phosphorylating), and state 4 (resting).

### Pravastatin Induces Mitochondrial Oxidative Stress in Treated *LDLr*^-/-^ Mice

**FIGURE 2:**
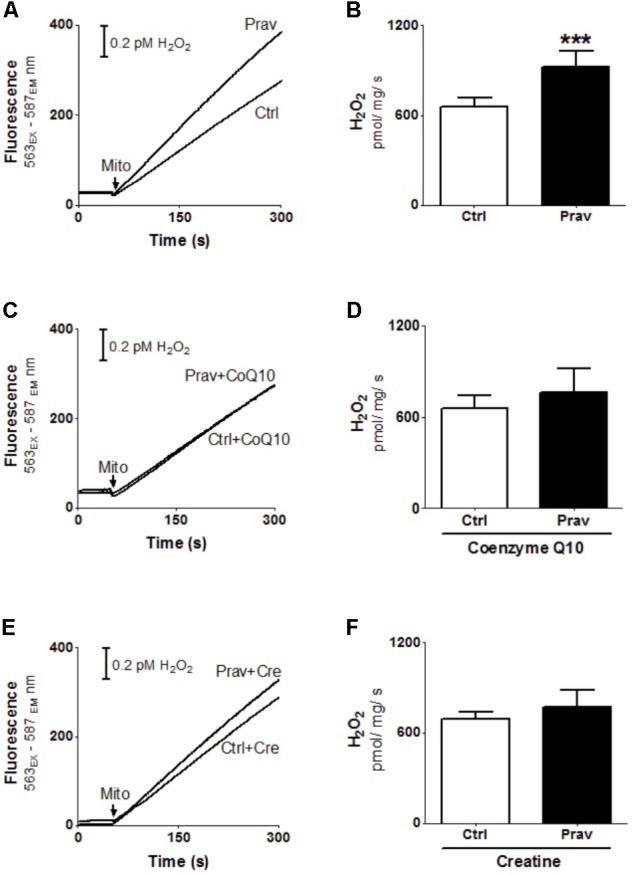
Pravastatin induces higher mitochondrial H_2_O_2_ generation that is neutralized by diet supplementation with CoQ10 or creatine in *LDLr*^-/-^ mice. H_2_O_2_ generation was monitored in liver mitochondria from *LDLr*^-/-^ mice treated or not with pravastatin **(A,B)**, and supplemented with CoQ10 **(C,D)** or creatine **(E,F)**. Mice liver mitochondria (MLM, 0.5 mg/mL) were added to standard medium in the presence of 10 μM Amplex-red and 1 U/mL horseradish peroxidase. Values are means ± standard deviation, eight independent experiments. ^∗∗∗^*P* < 0.001 compared to control (Mann–Whitney test).

**FIGURE 3:**
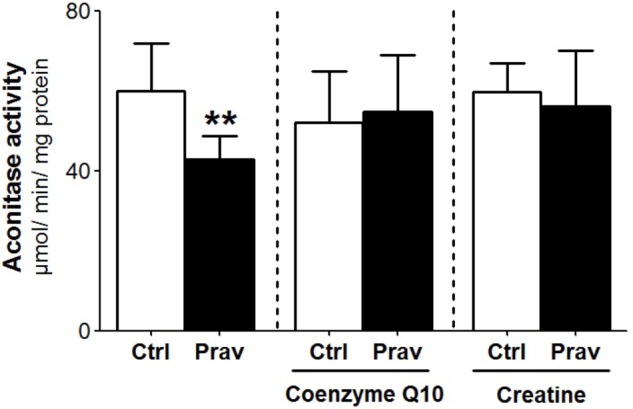
Pravastatin decreases aconitase activity and CoQ10 or creatine diet supplementation reverses this effect. Aconitase activity was evaluated in isolated liver mitochondria from control and pravastatin treated *LDLr*^-/-^ mice and supplemented with CoQ10 or creatine. Values are means ± standard deviation, six independent experiments and expressed as μmol/min/mg protein. ^∗∗^*P* < 0.01 compared to control (Mann–Whitney test).

Reactive oxygen production rates were measured in the presence of 200 μM EGTA. Hydrogen peroxide production was measured using the Amplex-red specific probe (**Figures 2A,B**). Pravastatin treatment promoted a higher H_2_O_2_ generation (40%) compared to mitochondria of control untreated mice (*P* < 0.001), as indicated by increased resorufin (Amplex-red derived product) fluorescence. To confirm the presence of elevated reactive oxygen species, the activity of aconitase, a Fe–S enzyme highly sensitive to superoxide (Gardner, 2002) was also evaluated (**Figure 3**). Pravastatin treatment decreased aconitase activity by 28% in liver mitochondria (*P* < 0.01) of pravastatin treated mice.

### CoQ10 or Creatine Diet Supplementation Abolishes the Pravastatin-Induced Reactive Oxygen Generation

Coenzyme Q10, that present antioxidant and anti-inflammatory properties (Sohet et al., 2009) is depleted in plasma and other tissues after statins treatments (Tavintharan et al., 2007; La Guardia et al., 2013). Here we show that diet supplementation with 0.3% of CoQ10 decreased mitochondrial H_2_O_2_ generation (**Figures 2C,D**) and restored aconitase activity in pravastatin treated group (**Figure 3**). To emphasize the importance of the oxidative stress as the main mechanism of pravastatin side effect, we supplemented mice diet with another mevalonate pathway independent antioxidant, creatine, which was recently shown to protect against muscle mitochondrial oxidative damage induced by pravastatin (Busanello et al., 2017). Indeed, **Figures 2E,F** show that creatine diet supplementation also significantly decreased H_2_O_2_ production and restored aconitase activity that were altered by pravastatin treatment (**Figure 3**).

### Ca^2+^-Induced Mitochondrial Permeability Transition Promoted by Pravastatin Is Prevented by CoQ10 or Creatine Supplementation

**FIGURE 4:**
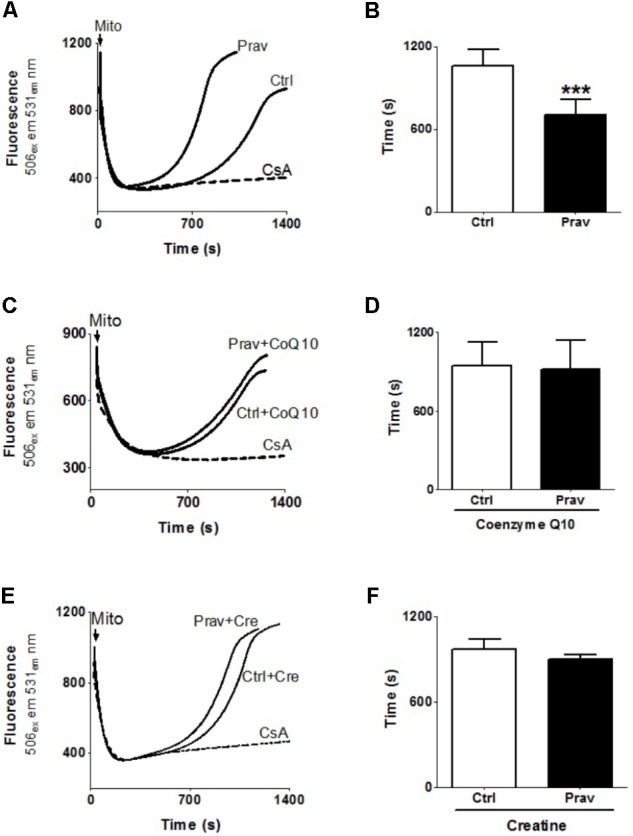
Pravastatin increases mitochondria susceptibility to Ca^2+^-induced membrane permeability transition that is prevented by diet supplementation with CoQ10 or creatine. Ca^2+^ uptake and retention capacity was evaluated in liver mitochondria from control and pravastatin treated *LDLr*^-/-^ mice **(A,B)** and supplemented with CoQ10 **(C,D)** or creatine **(E,F)**. Mice liver mitochondria (MLM, 0.5 mg/mL) were added to standard medium in the presence of 15 μM Ca^2+^ and 0.1 μM calcium green –5N. Values are means ± standard deviation of time elapsed to release calcium, eight independent experiments. ^∗∗∗^*P* < 0.001 compared to control (Mann–Whitney test).

Consistent literature data show that a combination of high Ca^2+^ levels in the mitochondrial matrix and reactive oxygen generation trigger the mitochondrial permeability transition (MPT) (Kowaltowski et al., 2001). To ascertain whether pravastatin could induce this effect in liver mitochondria, we investigated the susceptibility to Ca^2+^-induced MPT by following the changes in mitochondrial Ca^2+^ retention capacity in the presence or absence of EGTA and cyclosporine A (an MPT inhibitor). **Figures 4A,B** show that pravastatin treatment promoted a higher susceptibility to MPT (33%, *P* < 0.001) as evidenced by the shorter time taken by mitochondria from pravastatin treated mice to release calcium. **Figure 4** also shows that CoQ10 (**Figures 4C,D**) or creatine (**Figures 4E,F**) diet supplementation prevented MPT in the liver mitochondria of pravastatin treated *LDLr*^-/-^ mice.

### Pravastatin Treatment Upregulates Liver Glucose-6-Phosphate Dehydrogenase (G6PD) Activity

**FIGURE 5:**
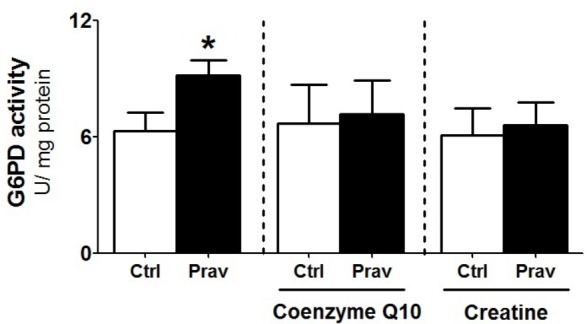
Pravastatin increases glucose-6-phosphate dehydrogenase (G6PD) activity that is abolished by CoQ10 or creatine diet supplementation. Enzyme activity was measured in liver homogenates from control and pravastatin treated *LDLr*^-/-^ mice and supplemented with CoQ10 or creatine. Values are means ± standard deviation, four independent experiments. ^∗^*P* < 0.02 compared to control (Mann–Whitney test).

Considering that several studies suggest that statins exert antioxidant action via upregulation of cellular antioxidant systems (Zhou and Liao, 2010; Busanello et al., 2017), we evaluated enzymatic antioxidant systems in *LDLr*^-/-^ mice liver homogenates. Pravastatin treatment caused no alterations in the activities of GR, GPx, Prx, SOD, and CAT (**Supplementary Table S1**). However, a 44% increase in the activity of G6PD, a cytosolic enzyme that generates NADPH, was observed in liver of pravastatin treated mice (**Figure 5**). CoQ10 or creatine diet supplementation that protected against mitochondrial dysfunction also abolished the pravastatin effect on G6PD activity (*P* < 0.02).

Since this enzyme participates in the cytosolic maintenance of NADPH levels (Yin et al., 2012), we also evaluated mitochondrial enzymes activities linked to NADP^+^ reduction. No alterations were found in the activities of isocitrate dehydrogenase-2, malic enzymes and glutamate dehydrogenase activities in liver mitochondria of both groups of *LDLr*^-/-^ mice (**Supplementary Table S1**).

### Pravastatin Increased GSH/GSSG Ratio in Liver Homogenates

**FIGURE 6:**
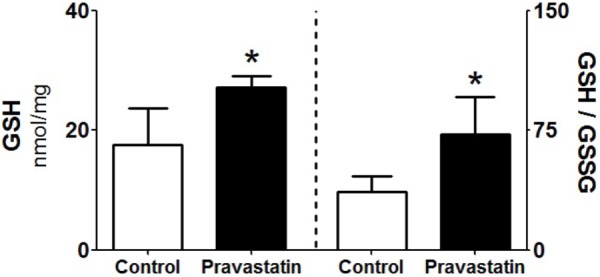
Pravastatin increases glutathione levels in liver homogenates. The reduced glutathione content (GSH) and reduced to oxidized glutathione ratio (GSH/GSSG) were determined. Values are means ± standard deviation, five independent experiments and expressed as nmol/mg protein. ^∗^*P* < 0.01 compared to control (Mann–Whitney test).

Since GSH is one of the most abundant reducing power in the hepatocyte (Yuan and Kaplowitz, 2009), we evaluated the levels of reduced and oxidized forms of glutathione in liver homogenates. A 53% increase in reduced glutathione levels (GSH) and twofold increase in GSH/GSSG ratio were observed in liver homogenate of pravastatin treated mice (**Figure 6**). No differences were observed in the liver content of NADPH (control: 0.25 ± 0.04 and pravastatin: 0.25 ± 0.05 nmol/mg protein) and NADP^+^ (control: 0.08 ± 0.00 and pravastatin: 0.06 ± 0.01 nmol/mg protein) of both groups of mice.

### Pravastatin Induced Lipid but Not Protein Oxidative Damage in Liver

Next, we searched for markers of pravastatin-induced oxidative damage to macromolecules (lipids and proteins). Using survey scan data from mass spectrometry (ESI-HRMS), the liver amphipathic lipid species profile of mice treated or not with pravastatin were compared. Differences in the lipid profiles were detected by orthogonal partial least squares discriminant analysis (OPLS-DA) and thereby confirmed that both groups are clearly separated. Permutation tests validated the model, with *P*-values of prediction accuracy during training (*P* = 0.05) and separation distance (*P* = 0.04) within the significance threshold (**Supplementary Figure S2**). Lipid markers in liver homogenates were identified with the assistance of Lipid MAPS database and are shown in **Table 1**. Notably, dominant chemical markers of pravastatin treated mice are oxidized lipids (7 out of 10 species) as compared to control (3 out of 10 species). Thus, oxidized lipids are more prevalent in liver from the pravastatin treated group, indicating the presence of an oxidative insult.

**Table 1:** Lipid markers identified by electrospray ionization high-resolution mass spectrometry in liver from *LDLr*^-/-^ mice.

Liver	Theoretical mass	Experimental mass	Error (PPM)	Lipid Class	Molecule ([M-H]^-^)
Control	333.2071	333.2075	-1.2	Docosanoid	Resolvin E2
	425.2545	425.2551	-1.4	Eicosanoid	Glyceryl PGD2
	387.2177	387.2183	-1.5	Eicosanoid	17-phenyl-trinor-PGF2α
	349.2021	349.2028	-2.0	Eicosanoid	Prostaglandin I3
	331.1915	331.1919	-1.2	Eicosanoid	Prostaglandin J3
	393.2283	393.2288	-1.3	Oxygenated docosanoid	PGF4α + O
	361.2390	361.2397	-1.9	Oxygenated fatty acid	Docosapentaenoic acid + O_2_
	359.2228	359.2229	-0.3	Oxygenated fatty acid	Docosahexaenoic acid + O_2_
	345.2799	345.2806	-2.0	Sterol lipid	24-Nor-5β-chol-22-ene-diol
	439.3582	439.3590	-1.8	Sterol lipid	32-oxolanosterol
Pravastatin	375.2182	375.2183	-0.3	Docosanoid	Resolvin D1/D2/D3
	493.2572	493.2581	-1.8	Eicosanoid	Leukotriene D5
	298.1061	298.1063	-0.7	Glycerophospholipid	1-acetylphosphocholine
	373.2020	373.2027	-1.9	Oxygenated docosanoid	17- or 8-oxo-Resolvin D1
	339.2541	339.2547	-1.8	Oxygenated fatty acid	C20:2 + O_2_
	313.2384	313.2390	-1.9	Oxygenated fatty acid	C18:1 + O_2_
	239.1653	239.1655	-0.8	Oxygenated fatty acid	C14:2 + O
	271.1915	271.1920	-1.8	Oxygenated fatty acid	C15:1 + O_2_
	285.2071	285.2075	-1.4	Oxygenated fatty acid	C16:1 + O_2_
	469.2208	469.2216	-1.7	Oxygenated glycerophosphocholine	C14:1 + O


*Structure assignment was based on mass accuracy of ions elected by OPLS-DA. The experimental mass is obtained for the ionized molecule and compared to the closest mass listed in the Lipid Maps database (http://www.lipidmaps.org). OPLS-DA, orthogonal partial least squares discriminant analysis; *n* = 10/group; PPM, parts per million.*

Additionally, we measured the content of protein carbonyl and free sulfhydryl groups in liver homogenates. Pravastatin treatment did not induce oxidative protein damage as indicated by similar protein carbonylation levels (control: 0.59 ± 0.05 and pravastatin: 0.58 ± 0.03 nmol/mg protein) and free sulfhydryl content (control: 9.79 ± 0.54 and pravastatin: 9.57 ± 0.69 nmol/mg protein).

## Discussion

Literature data on statins effects other than cholesterol lowering include antioxidant (Parihar et al., 2012), anti-inflammatory (Liao, 2002), anti-tumorigenic properties, and (Vallianou et al., 2014) improvement of the endothelial functions (Zhou and Liao, 2010). On the other hand, pro-oxidant (Kwak et al., 2012) and diabetogenic actions (Lorza-Gil et al., 2016; Urbano et al., 2017) are also reported. Regarding mitochondria, it has been shown that statins induce inhibition of mitochondrial respiration (La Guardia et al., 2013), mitochondrial oxidative stress and lipid peroxidation (Velho et al., 2006; Oliveira et al., 2008; Abdoli et al., 2013; Costa et al., 2013), imbalance in calcium homeostasis (Sirvent et al., 2005), cytochrome c release, DNA fragmentation, and β-oxidation inhibition (Kaufmann et al., 2006). These effects are attributed mainly to lipophilic statins whereas the hydrophilic ones seem to be less toxic (Velho et al., 2006; Tolosa et al., 2015; Busanello et al., 2017; Urbano et al., 2017).

Taking into account that liver is the major steroidogenic organ and thus a main target of these drugs (Chatzizisis et al., 2010), data on statins hepatotoxicity are scarce. Here, we present the results of a chronic treatment of a hypercholesterolemic mice model with a hydrophilic pravastatin that induced liver mitochondria dysfunction. We found that pravastatin treated mice presented increased mitochondrial H_2_O_2_ production rates, reduced aconitase activity and increased susceptibility to MPT. Accordingly, we have recently reported that plantaris muscle from the same pravastatin treated mice model presented mild mitochondrial oxidative stress and higher sensitivity to Ca^2+^-induced MPT (Busanello et al., 2017). Here, we observe that liver seems to be more resistant to the side effects than skeletal muscle, since pravastatin did not inhibit mitochondrial respiration. In liver, we also observed that pravastatin decreased mitochondrial aconitase activity, a very sensitive target of superoxide attack that was not observed in muscle (Busanello et al., 2017). In addition, we found liver oxidized lipid species but not oxidized protein markers in pravastatin treated mice. Therefore, the main mechanism of pravastatin hepatotoxicity is the induction of a mild mitochondrial oxidative stress. This is confirmed by the fully prevention of both reactive oxygen generation and MPT by the *in vivo* supplementation with two distinct antioxidants, CoQ10 and creatine.

The rational to use CoQ10 diet supplementation was to investigate its *in vivo* efficacy against the mitochondrial oxidative stress, since most previous studies had shown only *in vitro* CoQ10 action (La Guardia et al., 2013; Tolosa et al., 2015; Busanello et al., 2017; Sadighara et al., 2017) and a reasonable doubt on effectiveness of oral CoQ10 supplementation persists (Taylor, 2017). The present results clarify two major points on the mechanism of CoQ10 protection against statins side effects: first, administration of CoQ10 suppresses the endogenous deficiency of this coenzyme promoted by statins inhibition of its biosynthesis (HMG-CoA reductase pathway). In fact, the depletion of this coenzyme was observed in the liver of our experimental model (Lorza-Gil et al., *unpublished data*) as well as in skeletal muscle, heart, brain, plasma, HepG2 and insulin-secreting INS-1 cells after statins treatments (Tavintharan et al., 2007; La Guardia et al., 2013; Urbano et al., 2017). The second point is that CoQ10 effects are mediated mostly by its free radical scavenger action (Tavintharan et al., 2007; Eghbal et al., 2014) rather than by its electron transfer function, since under the present conditions, pravastatin did not inhibit mitochondria respiration. This mechanism was previously proposed by our group (La Guardia et al., 2013; Busanello et al., 2017).

The diet supplementation with creatine, a compound endogenously synthesized (independently of the HMGCoA reductase pathway) and obtained from the diet, also protected against oxidative stress and Ca^2+^-induced MPT in the liver, as observed previously in skeletal muscle of pravastatin-treated *LDLr*^-/-^ mice (Busanello et al., 2017). Creatine administration was previously shown to protect against hypoxia, ischemia, neurodegeneration and oxidative damage (Rambo et al., 2013). Literature data suggest that these beneficial effects of creatine could be attributed to its direct antioxidant properties, demonstrated by its ability to remove superoxide and peroxynitrite (Lawler et al., 2002; Guimarães-Ferreira et al., 2012), or to its function as substrate for the mitochondrial creatine kinase (mCK) (Wallimann et al., 2011). The mCK mediates the reversible interconversion of creatine and ATP into phosphocreatine, an energy donor that under challenging conditions can leave mitochondria and supply the cytosolic ATP demand (Wallimann et al., 2011). Since mice do not express mCK in liver (Miller et al., 1997), the present data strongly support the free radical scavenger hypothesis to explain the protection against pravastatin induced oxidative stress. The present results also suggest that endogenously generated creatine in these hypercholesterolemic mice was insufficient, since exogenous creatine supplementation was required for effective protection.

Another interesting observation is the increased G6PD activity in the liver of pravastatin treated mice. G6PD is a cytosolic enzyme that regenerates NADPH, the main reducing power used to sustain H_2_O_2_ detoxification by the glutathione system. Thus, G6PD is considered a major enzyme for the cellular defense against oxidative stress (Pandolfi et al., 1995) and its activity is strongly modulated in response to oxidants (Kletzien et al., 1994). Indeed, increased G6PD activity was also found in liver of rats treated with rosuvastatin and in adipose tissue of rats treated with atorvastatin or lovastatin (Aguirre et al., 2013). The increase of G6PD activity (observed in liver but not in muscle of pravastatin treated mice) may be considered a compensatory mechanism to provide extra NADPH to reduce GSSG to GSH, the most abundant reducing agent in liver (Yuan and Kaplowitz, 2009). Although a higher level of G6PD derived NADPH in the pravastatin group would be expected, its rapid consumption to regenerate GSH may explain similar liver levels in both groups. Besides maintaining the redox state of the protein sulfhydryl groups, GSH also quenches endogenous and exogenous oxidant species (Yuan and Kaplowitz, 2009). In fact, the higher levels of GSH in liver seems to be part of an adaptive response to the pravastatin induced mitochondrial oxidative stress.

## Conclusion

In conclusion, this study demonstrates for the first time in the appropriated biological context (hypercholesterolemia) that chronic pravastatin treatment leads to liver mitochondrial redox imbalance and Ca^2+^-induced MPT. These findings may explain the hepatic side effects reported in a small number of patients. The protection exerted by co-treatment with two distinct antioxidants, CoQ10 or creatine, reinforce that pravastatin hepatotoxicity is caused by an oxidative stress. These safe antioxidants may be used to neutralize pravastatin side effects.

## Author Contributions

AM and EB conducted the experiments, analyzed the data, and wrote the manuscript. DdO and RC performed the lipidomic analyses and interpretation. HO and AV were co-responsible for obtaining the grants, project conception, research design, data analyses, and manuscript writing.

## Conflict of Interest Statement

The authors declare that the research was conducted in the absence of any commercial or financial relationships that could be construed as a potential conflict of interest.

## Supplementary Material

FIGURE S1 Pravastatin treatment did not affect liver histology of *LDLr*^-/-^ mice. Representative images of **(A)** Hematoxylin and eosin (HE), **(B)** Masson’s trichrome (MT), and **(C)** picrosirus red (PSR) staining. Scale bar represent 50 μm (original magnification ×40).

FIGURE S2 Lipidome scores plot obtained with orthogonal partial least squares discriminant analysis (OPLS-DA). Permutation tests validated the model of liver lipidome, with *P*-values of prediction accuracy during training (*P* = 0.05) and separation distance (*P* = 0.04) within the significance threshold.  treated with pravastatin and  control.

TABLE S1 Liver antioxidant enzymes and mitochondrial NADP-dependent enzymes activities in pravastatin treated *LDLr*^-/-^ mice.
