# Supplementary figures and images for: Coenzyme Q10 or Creatine Counteract Pravastatin-Induced Liver Redox Changes in Hypercholesterolemic Mice
Source: Front Pharmacol. 2018 Jun 27;9:685. doi: 10.3389/fphar.2018.00685 (PMC6030358; doi:10.3389/fphar.2018.00685)

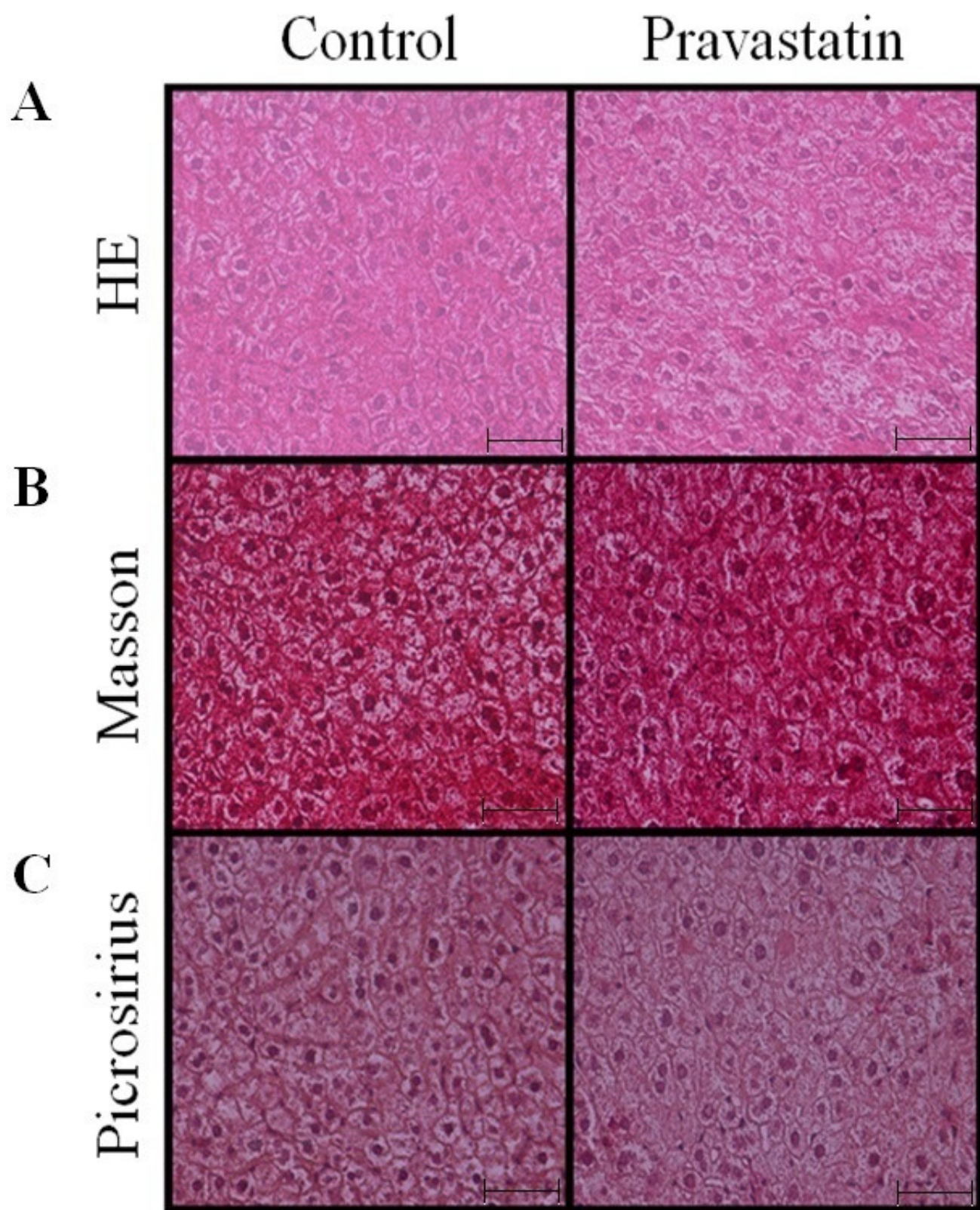

Supplement: FIGURE S1 — Pravastatin treatment did not affect liver histology of LDLr-/- mice. Representative images of (A) Hematoxylin and eosin (HE), (B) Masson’s trichrome (MT), and (C) picrosirus red (PSR) staining. Scale bar represent 50 μm (original magnification ×40). [file Image_1.PDF]

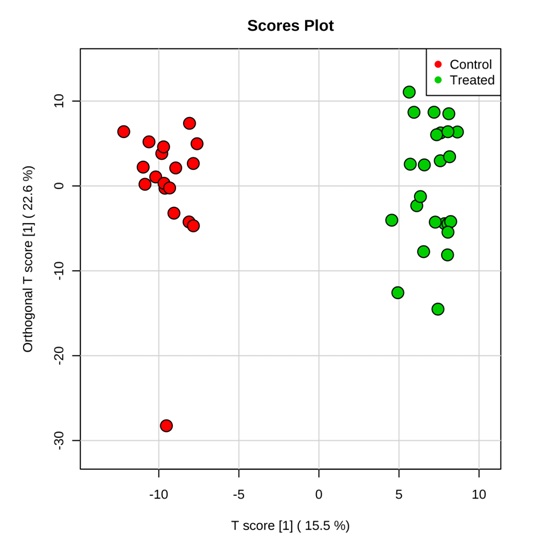

Supplement: FIGURE S2 — Lipidome scores plot obtained with orthogonal partial least squares discriminant analysis (OPLS-DA). Permutation tests validated the model of liver lipidome, with P-values of prediction accuracy during training (P = 0.05) and separation distance (P = 0.04) within the significance threshold. treated with pravastatin and control. [file Image_2.JPEG]
