# Supplementary material for: Coenzyme Q10 or Creatine Counteract Pravastatin-Induced Liver Redox Changes in Hypercholesterolemic Mice
Source: Front Pharmacol. 2018 Jun 27;9:685. doi: 10.3389/fphar.2018.00685 (PMC6030358; doi:10.3389/fphar.2018.00685)
Supplement: TABLE S1 — Liver antioxidant enzymes and mitochondrial NADP-dependent enzymes activities in pravastatin treated LDLr-/- mice. [file Table_1.docx]

**Supplementary Table I: Liver Antioxidant enzymes and mitochondrial NADP-dependent enzymes activities in pravastatin treated *LDLr*-/- mice.**

| \|  \|  \| **Control** \| **Pravastatin** \| \| --- \| --- \| --- \| --- \| \|  \| **GR** \| 7.09 ± 0.53 \| 8.33 ± 0.43 \| \| Total Liver Antioxidant enzymes \| **GPx** \| 22.21 ± 1.17 \| 19.90 ± 1.77 \| \| **SOD** \| 19.40 ± 1.49 \| 17.65 ± 0.49 \| \| **CAT** \| 11.40 ± 0.95 \| 12.12 ± 1.07 \| \| **Prx** \| 24.73 ± 2.39 \| 20.20 ± 3.39 \| \| Liver mitochondria NADP-dependent enzymes \| **IDH** \| 59.64 ± 7.15 \| 70.95 ± 4.98 \| \| **ME** \| 26.30 ± 3.97 \| 18.12 ± 1.34 \| \| **GDH** \| 18.66 ± 1.16 \| 18.12 ± 1.34 \| |
| --- | --- | --- | --- | --- | --- | --- | --- | --- | --- | --- | --- | --- | --- | --- | --- | --- | --- | --- | --- | --- | --- | --- | --- | --- | --- | --- | --- | --- | --- | --- | --- |

GR, glutathione reductase; GPx, glutathione peroxidase; Prx, peroxiredoxin; SOD, superoxide dismutase; CAT, catalase; IDH, isocitrate dehydrogenase-2; ME, malic enzymes; GDH, glutamate dehydrogenase activities. The liver antioxidant and mitochondrial NADP-dependent enzymes activities are expressed as U/mg protein and mU/mg protein, respectively. Values are means ± standard deviation, five independent experiments. No significant differences were observed (Mann–Whitney test).
